# Placental Growth Factor and Female Long-Term Hypertension

**DOI:** 10.3390/jcm14196751

**Published:** 2025-09-24

**Authors:** Maria C. Adank, Jeanine E. Roeters Van Lennep, Laura Benschop, James M. Roberts, Robin E. Gandley, Yolanda B. De Rijke, Eric A. P. Steegers, Sarah Schalekamp-Timmermans

**Affiliations:** 1 Department of Obstetrics and Gynaecology, Erasmus MC, University Medical Centre Rotterdam, P.O. Box 2040, 3000 CA Rotterdam, The Netherlands; 2 Generation R Study Group, Erasmus MC, University Medical Centre Rotterdam, P.O. Box 2040, 3000 CA Rotterdam, The Netherlands; 3 Department of Internal Medicine, Erasmus MC, University Medical Centre Rotterdam, P.O. Box 2040, 3000 CA Rotterdam, The Netherlands; 4 Department of Obstetrics and Gynecology and Reproductive Sciences, Magee-Womens Research Institute, University of Pittsburgh, Pittsburgh, PA 15213, USA; 5 Department of Epidemiology and Clinical and Translational Research, University of Pittsburgh, Pittsburgh, PA 15213, USA; 6 Department of Clinical Chemistry, Erasmus MC, University Medical Centre Rotterdam, P.O. Box 2040, 3000 CA Rotterdam, The Netherlands

**Keywords:** placental growth factor, cardiovascular disease, hypertension, females

## Abstract

**Background and Aims:** Placental growth factor (PlGF) is an important predictive marker of pregnancy complications such as preeclampsia. The aim of this study is to assess whether PlGF measured outside of pregnancy is a predictive marker for cardiovascular disease (CVD) risk in young women. **Methods:** This study was embedded in the Generation R Study, a population-based prospective cohort study. PlGF concentrations, as well as systolic and diastolic blood pressure (SBP and DBP), cardiac outcomes, carotid-femoral pulse wave velocity, and central retinal arteriolar and venular calibres of 5077 women, were assessed six years after pregnancy, which was considered baseline. Four years after baseline, we measured blood pressure and intimal media thickness (IMT). Eight years after baseline, we measured blood pressure and the post-occlusive reactive hyperaemia index (PORH index). In addition, we examined the influence of pregnancy complications on these associations. **Results:** We found a positive association between PlGF levels with SBP (0.46, 95% CI 0.04; 0.89). PlGF was not associated with retinal or echocardiographic measurements. PlGF was positively associated with DBP after four years and with both SBP and DBP eight years after baseline, independent of the occurrence of pregnancy complications. PlGF was not associated with IMT or the PORH index. **Conclusions:** PlGF is associated with higher blood pressure. PlGF may, therefore, be used as a marker of hypertension. These results need to be replicated in an independent cohort study.

## 1. Introduction

Placental growth factor (PlGF) is a member of the vascular endothelial growth factor family and was originally found to promote angiogenesis in placental tissue [1]. During pregnancy, the syncytiotrophoblast decreases the production of PlGF in the maternal circulation in response to stress [2]. Women with reduced PlGF concentrations in pregnancy are at increased risk of developing pregnancy complications, such as preeclampsia (PE) and spontaneous preterm birth (sPTB), and of giving birth to a child born small-for-gestational age (SGA), also called placental syndromes [3]. Women with a placental syndrome are at increased risk of cardiovascular disease (CVD) later in life [4]. A recent study performed in our study cohort found that women with lower PlGF concentrations in mid-pregnancy had an increased cardiovascular risk six years after pregnancy compared to women with higher concentrations of PlGF at mid-pregnancy [5].

PlGF is not only expressed in placental tissue. Outside of pregnancy, it is also expressed by other cell types, including endothelial cells, in both men and women. PlGF affects several cell types and plays a role in inflammation, cancer, wound healing, and bone fracture healing [6,7,8,9,10,11,12,13]. It also plays a role in a wide variety of chronic diseases, ranging from cancer to colitis, sepsis, and liver cirrhosis [14]. Interestingly, PlGF seems redundant in developmental and physiological processes but is more important in settings of disease with vascular damage [14,15,16,17,18]. Previous studies have shown that elevated PlGF may act as an independent biomarker of short- and long-term adverse outcomes in patients with acute coronary syndrome [8,19] and as an independent predictor of cardiovascular disease (CVD) in patients with diabetes mellitus type 1 [20,21]. Moreover, PlGF may play a role in the pathogenesis of atherosclerotic disease [22]. The role of PlGF outside of pregnancy in a healthy population remains unclear. We hypothesised that, as with PlGF levels in pregnancy, PlGF levels measured six years after an index pregnancy may be a useful predictor of cardiovascular risk in women. Therefore, the aim of this study was to assess the association between PlGF concentration six years after pregnancy and blood pressure, cardiac outcomes, and cardiovascular health years later. In addition, we examined the influence of pregnancy complications on these associations.

## 2. Materials and Methods

### 2.1. Study Design

**Figure 1:**
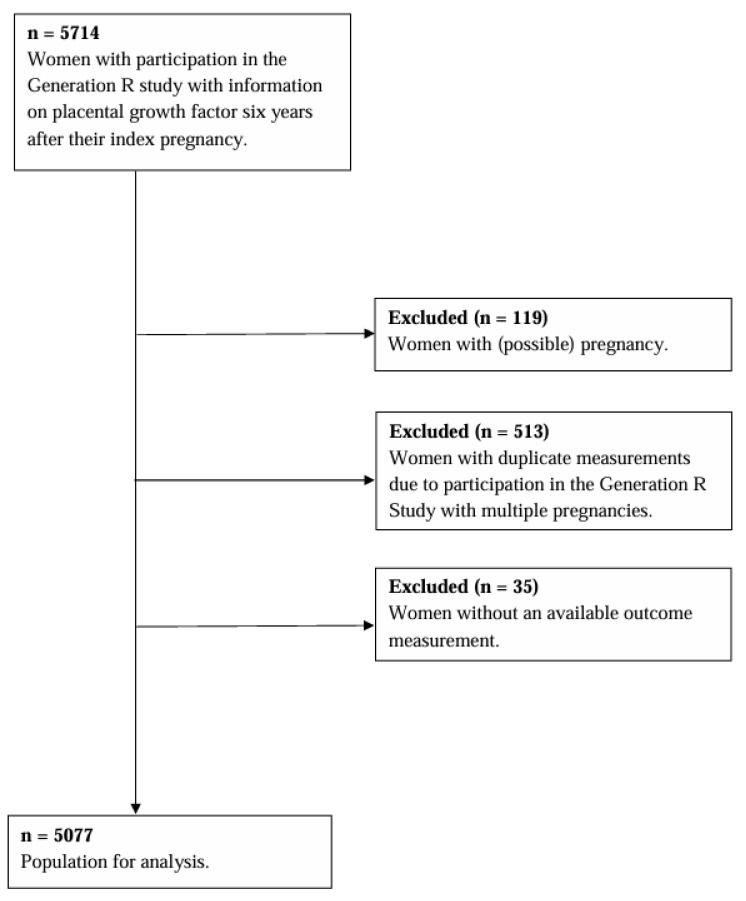
Flowchart.

This study was embedded in the Generation R Study, a population-based prospective cohort from early pregnancy onwards [23,24]. All pregnant women living in Rotterdam with an expected delivery date between April 2002 and January 2006 were eligible for participation. All women were invited to standardised visits at the research centre during pregnancy and years after their pregnancy. Written informed consent was obtained from all participants. We included women who had PlGF measurements six years after their index pregnancy [5] and considered this the baseline for this study. Women with a possible pregnancy at the time of blood sampling or women with duplicate measurements due to participation in the Generation R Study with multiple pregnancies were excluded. For the present study, we included 5077 women (Figure 1). The Medical Ethics Committee of the Erasmus Medical Centre Rotterdam, the Netherlands, approved the start of the Generation R Study on 23 January 2001 (MEC 198.782/2001/31). The data used in this study were collected in the follow-up period. Ethical approval was granted by the Medical Ethics Committee of Erasmus Medical Centre on 15 December 2015 (MEC-2015-749 NL 55105.078.15).

### 2.2. Exposure: PlGF Concentration

Our exposure was PlGF measured six years after an index pregnancy (median 6.0 years; 90% range, 5.7; 7.3), and we considered this timepoint the baseline. All samples were taken by trained research nurses and stored at the research facility at room temperature for a maximum of three hours, after which they were sent to the laboratory facility [24]. PlGF concentrations were measured on a cobas e 801 analyser (Roche Diagnostics, Mannheim, Germany). The inter-laboratory coefficients of variation were below 4%. A functional sensitivity (20% CV) of less than 4 pg/mL was calculated from all QC data.

### 2.3. Outcomes and Covariates at Baseline

At baseline, we obtained information on maternal age, ethnicity, educational level, gravidity, smoking, and medication use through questionnaires. We measured maternal height (cm) and weight (kg) without shoes and calculated BMI (kg/m^2^). Blood pressure was measured twice in the sitting position with the validated automatic sphygmomanometer Datascope Accutorr Plus (Paramus, NJ, USA). The average of both blood pressure measurements was used for further analyses. Carotid-femoral pulse wave velocity (PWV) was measured with an automatic non-invasive, validated device (Complior^®^; Artech Medical, Pantin, France) to assess arterial wall stiffness. We performed two-dimensional M-mode echocardiographic measurements using the ATL-Philips Model HDI 5000 (Seattle, WA, USA) or the Logiq E9 (GE Medical Systems, Wauwatosa, WI, USA) devices. Fractional shortening (FS), aortic root diameter (AOD), and left atrial diameter (LAD) were measured. Left ventricular (LV) mass was calculated using the equation derived by Devereux [25]. We took unilateral digital retinal photographs of maternal retinal vascular calibres of the left eye using a Topcon digital retinal camera (model TRC, NW300) with image resolutions set to 4096 and 3072 pixels [26].

### 2.4. Outcomes Four Years After Baseline

Blood pressure was measured in a supine position with the validated automatic sphygmomanometer Datascope Accutorr Plus (Paramus, NJ, USA). BMI (kg/m^2^) was calculated from maternal height (cm) and weight (kg) without shoes. The common carotid artery (CCA) was measured using the ATL-Philips Model HDI 5000 (Seattle, WA, USA) or the Logiq E9 (GE Medical Systems, Wauwatosa, WI, USA) device. Intima media thickness (IMT) was assessed with the subjects supine, with the head tilted slightly to the contralateral side for the measurement in the CCA. The analyses were performed offline and semi-automatically, using the application Carotid Studio (Cardiovascular Suite (Quipu srl, Pisa, Italy)). IMT was computed as the mean of three successive recordings from both the left and right sides.

### 2.5. Outcomes Eight Years After Baseline

Blood pressure was measured with the validated automatic sphygmomanometer Datascope Accutorr Plus (Paramus, NJ, USA) with participants supine. Sustained hypertension at fourteen years follow-up was defined when women used antihypertensive medication or if they had a mean SBP ≥ 140 mmHg and/or a DBP ≥ 90 mmHg during their visit to the research centre. Post-occlusive reactive hyperaemia (PORH) was measured with a laser probe (DP1T-V2 Skin Probe, Moor Instruments, Axminster, UK) fixed on the volar surface of the left antebrachium, distal to a sphygmomanometer occlusion cuff placed around the left upper arm 1–2 cm above the antecubital crease. The probe was lightweight and was attached to the skin by double-sided adhesive tape to reduce gross variation in pressure. Stable baseline skin perfusion flux was recorded for 3 min. Thereafter, the occlusion of the brachial artery was performed for 3 min by inflating the occlusion cuff to suprasystolic pressure. Immediately after decompression of the occlusion cuff, the reactive hyperaemia perfusion flux changes were recorded for three minutes. Changes in skin blood perfusion flux were analysed off-line using MoorSoft for Windows software package version 4.1 (Moor Instruments). We determined the PORH index, which is defined as the area under the curve one minute after release/the area under the curve one minute before inflation of the cuff.

### 2.6. Pregnancy

Placental syndromes included pregnancies complicated by pre-eclampsia (PE), a spontaneous preterm birth (sPTB), or a child born small-for-gestational age (SGA). We obtained information on clinically diagnosed PE from medical records that were cross-checked with the original hospital charts [27]. PE was defined, using the ISSHP criteria that were in effect at the time of the study, as new onset systolic blood pressure ≥ 140 mmHg and/or a diastolic blood pressure ≥ 90 mmHg after 20 weeks of gestation and the presence of proteinuria with no evidence of urinary tract infection in a random urine sample [28]. Midwife and hospital registries provided information on gestational age at birth, birth weight, and the child’s sex assigned at birth. SGA was defined as a child with a birth weight below the 10th percentile adjusted for gestational age and the sex assigned at birth of the child in our study cohort. We defined sPTB as the spontaneous onset of labour before 37 weeks of gestation.

### 2.7. Statistical Analyses

We examined baseline characteristics within the total population. To reduce potential bias due to missing data, we imputed missing values in covariates used as confounders in the regression analyses through multiple imputation procedures. Data were imputed according to the Markov Chain Monte Carlo method, assuming no monotone missing pattern. Data were analysed in each set separately, and pooled estimates from the five imputed datasets were used to report the effect estimates and their 95% confidence intervals. For the multiple imputation procedure, we performed 10 iterations [29]. At baseline, 2.3% had missing information on ethnicity, 14.4% on educational level, 28.0% on gravidity, 29.2% on smoking, and 10.1% on BMI. We tested whether the association of PlGF with corresponding cardiovascular outcomes was nonlinear. Since we found a linear relation, we further examined linear and logistic regression analyses to relate PlGF to cardiovascular markers at baseline and four and eight years later. Women using antihypertensive medication were excluded from the blood pressure analyses. The regression models include covariates selected based on their associations with the outcome of interest, based on previous studies or based on a change in the effect estimate of >10%. The basic model included the following variables, depending on the outcome of interest: maternal age at baseline, gravidity, ethnicity, educational level, smoking at baseline, pulse at the time of PWV measurement, and central retinal vascular calibre at baseline. Since BMI is associated with PlGF [30,31], the BMI model additionally included BMI at baseline. We repeated all analyses in women without placental syndromes during the index pregnancy (PE, SGA, or sPTB). Statistical Package of Social Sciences version 24.0 for Windows (IBM Corp., Armonk, NY, USA) was used.

## 3. Results

At baseline, the median PlGF concentration was 10.0 pg/mL (90% range, 6.0; 15.0 pg/mL).

Table 1 presents the maternal characteristics at baseline. Women were on average 37.3 years old, and most were European, highly educated, and had a median BMI of 24.4 (19.7; 35.3) kg/m^2^.

**Figure 2:**
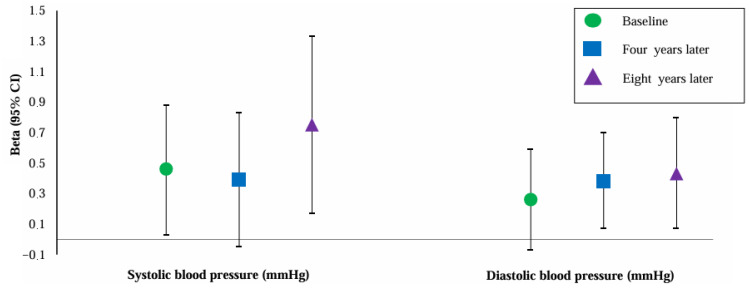
Association of placental growth factor with blood pressure at baseline and four and eight years later (n = 4963). Abbreviations: CI, confidence interval. Data were adjusted for maternal age, gravidity, ethnicity, educational level, smoking, and BMI at baseline. Values are linear regression coefficients (95% confidence interval).

Figure 2 shows the association of PlGF with SBP and DBP at baseline and four and eight years later. PlGF was positively associated with SBP at all timepoints. However, the association of PlGF with SBP four years after baseline became non-significant after adjustment for maternal BMI. PlGF was positively and independently associated with DBP four and eight years after baseline. PlGF was also positively associated with DBP at baseline, but these associations attenuated to non-significance if we adjusted for BMI.

PlGF measured at baseline was positively associated with LAD and LV mass; however, these associations were no longer significant if we adjusted for BMI (Table 2). Moreover, women with higher levels of PlGF had an increased risk of developing sustained hypertension eight years later (OR 1.16, 96% CI [1.01; 1.33]). No association of PlGF with the PORH index was found (Table 2).

### Placental Syndromes

In total, 756 (14.9%) women were classified as having a placental syndrome in their index pregnancy. PlGF measured at baseline was not different for women with or without a placental syndrome in their index pregnancy (medians, 10.0 pg/mL (95% CI 6.0; 16.0) and 10.0 pg/mL (95% CI 6.0; 14.6), respectively). After exclusion of women with placental syndromes, PlGF was still associated with SBP eight years later and DBP four and eight years after baseline (Table 3).

## 4. Discussion

Our study shows that PlGF levels are positively associated with blood pressure several years after baseline. PlGF was positively associated with SBP and DBP eight years later independent of the occurrence of placental syndrome in the index pregnancy.

PlGF concentrations increase substantially during pregnancy, from the level of nonpregnant women (<50 pg/mL) to levels exceeding 500 pg/mL after 30 weeks of gestation [32]. Women without an adequate increase in PlGF concentrations during pregnancy have a higher risk of developing placental syndromes, and they have an increased cardiovascular risk later in life [5,33,34,35]. Low circulating concentrations of PlGF are probably a consequence of abnormal placentation and may be a contributing factor to continued abnormal growth during pregnancy [33]. Outside of pregnancy, PlGF expression is low or undetectable in most healthy tissues but significantly upregulated in pathological conditions [14,17,18]. This agrees with our finding that PlGF outside of pregnancy is positively associated with blood pressure and may be used as a marker of cardiovascular health. These results need to be replicated in an independent cohort study.

In this study, PlGF was associated with blood pressure but not with echocardiographic measurements. There may be two explanations for this finding. First, it may be possible that the women in our study were too young to develop organ damage, and therefore, we only found an association with blood pressure. Second, it may be possible that the blood pressure that we found is still relatively low and, therefore, does not yet lead to echocardiographic changes. We assume that PlGF primarily affects endothelial function and, via this route, leads to increased blood pressure. Endothelial dysfunction decreases nitric oxide availability, leading to an elevation in blood pressure [36]. PlGF is mainly expressed by endothelial cells in pathological processes and has atherogenic properties caused by the recruitment and adhesion of monocytes, the production of proteolytic factors, and thrombus formation [37]. Surprisingly, we did not find an association of PlGF with the PORH index, which we assumed reflected endothelial function. In previous studies, PORH was lower with type 2 diabetes mellitus and in patients with coronary artery disease [38,39]. A recent study by Huri et al. found that higher concentrations of PlGF in pregnancy were associated with a higher post-occlusive reactive hyperaemic response [40]. A lower post-occlusive reactive hyperaemic response indicates impaired microvascular function and has been associated with cardiovascular diseases [41]. However, Huri et al. show only a moderate correlation of PlGF with PORH parameters, suggesting a limited impact on acute microvascular reactivity, which is consistent with the finding of no association of PlGF with retinal microvasculature in this study. Microvascular function may remain intact in early or mild forms of vascular dysfunction, especially if compensatory mechanisms are active [42,43]. This may explain why PlGF is associated with blood pressure changes but not with impaired PORH or retinal outcomes, indicating non-impaired microvascular function despite systemic vascular involvement.

In this study, PlGF was positively associated with LAD and LV mass, both associated with cardiovascular risk. These positive associations were not significant after adjustment for BMI. This indicates that BMI makes a substantial contribution to LAD and LV mass. It is known that LV mass markedly increases with the severity of obesity, which commonly leads to diastolic dysfunction [44,45]. In addition, diastolic dysfunction in the obese increases with increases in BMI [44,46]. Furthermore, hypertension has a negative impact on LAD and LV mass [47], and BMI is associated with hypertension [48]. Our study population was relatively healthy with a normal BMI; therefore, we could not investigate the association of PlGF with LAD and LV mass in a subgroup of obese women, including the impact of hypertension on these associations. Further research into these associations is needed.

To date, it is unclear whether the observations of an association between elevated PlGF and cardiovascular risk reflect activation of a protective response by a stressed vasculature or whether PlGF is pro-atherogenic. The pro-atherogenic potential of PlGF has been attributed to the ability to activate endothelial adhesion molecule expression and monocyte recruitment to the arterial wall [49,50,51]. Santalahti et al. reported that high baseline plasma PlGF predicted risk of cardiovascular mortality [52]. Additionally, Chen et al. also showed that this association is dependent on the presence of risk factors known to cause injury to the vascular wall [53]. It may be possible that this reflects the presence of a repair response activated by vascular stress rather than a pro-atherogenic role of PlGF, as would be consistent with the findings of Folkersson et al. regarding the potential protective role of PlGF in coronary heart disease [54]. However, the study by Folkersson et al. does not discriminate between men and women, and hypertension without coronary heart disease is not investigated. Therefore, it is difficult to extrapolate these findings to our study. Future studies should examine this association more closely.

There are at least four isoforms of PlGF [55,56,57]. The main difference between the isoforms is that PlGF-1 and PlGF-3 do not contain a heparan sulphate-binding domain and can potentially affect target cells in a paracrine manner, whereas PlGF-2 and PlGF-4 contain heparan sulphate-binding domains and most likely work in an autocrine way [55]. Proteoglycans on the luminal surface of endothelial cells contain heparan sulphate side-chains [58]; therefore, it is likely that PlGF-2 and PlGF-4 affect endothelial dysfunction through this association. PlGF could function to influence vascularity, as well as to influence trophoblast function, depending on the expressed isoform of PlGF. During pregnancy, the placenta produces and releases large amounts of PlGF into the maternal circulation. The major isoforms expressed in pregnancy are thought to be PlGF-1 and PlGF-2 [55]. Higher PlGF levels in pregnancy are associated with robust placental invasion and vascularisation. The absence of this response results in lower PlGF concentrations in mid-gestation. Both PlGF-1 and PlGF-2 concentrations are significantly decreased in pregnancies complicated by pre-eclampsia and SGA [59,60,61]. In addition, decreased concentrations of PlGF in mid-pregnancy are associated with an increased cardiovascular risk later in life [5]. Our current study indicates that higher concentrations of PlGF measured years after pregnancy, rather than lower concentrations, as found in pregnancy, are associated with an increased cardiovascular risk regardless of pregnancy history. Outside of pregnancy, PlGF rises in response to vascular challenges such as atherosclerotic lesions [51]. It may be possible that vascular damage decreases the availability of heparin sulphate-binding domains, resulting in higher levels of circulating PlGF. Future studies should focus on the different isoforms, bioavailability, and regulation of expression of PlGF [62].

Women with a placental syndrome have an increased risk of hypertension and cardiovascular disease later in life [4]. In this study, PlGF measured at baseline was no longer associated with SBP if we excluded women with placental syndromes. However, PlGF was still associated with blood pressure eight years later. This suggests that PlGF has more impact on blood pressure later in life.

### Strengths and Limitations

We collected prospective data on a large population of 5077 women with available blood samples. All outcomes were obtained following standardised protocols. Our population only contains women with a prior pregnancy resulting in a liveborn singleton. Unfortunately, in our study, we were not able to quantify the PlGF isoforms. Future studies should investigate PlGF isoforms and cross-reactivity to investigate its inverse role as a cardiovascular risk marker in pregnancy. Blood pressure measurements were standardised for all participants at the same timepoint; however, there was a difference in measurement between timepoints. At baseline, blood pressure was measured with participants sitting, while four and eight years after pregnancy, participants were supine during blood pressure measurements. The body posture of participants especially influences diastolic blood pressure, which is higher when sitting compared to standing [63]. This effect decreases with age but still may explain why blood pressure measurements taken with the women supine four years after baseline were lower than the measurements taken at baseline with the women seated. In women with hypertension, both SBP and DBP may be higher sitting than supine [64]. It may, therefore, be possible that we found an underestimation of the association of PlGF with blood pressure four and eight years after baseline. At each timepoint, all participants were measured the same way, which we considered a non-differential misclassification. The selection of a relatively healthy population affects the generalizability of the results to a higher-risk population. This is in line with other large population-based cohort studies [23,65]. The observational nature of this study does not allow for the inference of causality. Although we adjusted for numerous potential confounders, residual confounding caused by other lifestyle factors might still be present.

## 5. Conclusions

The results of this study suggest that PlGF levels are a predictive marker of blood pressure in relatively young women. Years after pregnancy, higher concentrations of PlGF, rather than lower concentrations, as found in pregnancy, are associated with increased blood pressure. Therefore, PlGF may not only be used as a predictive marker of cardiovascular health in pregnancy but also, outside of pregnancy, as a marker of hypertension. These results should be replicated in an independent cohort study.

## Figures and Tables

**Table 1:** Baseline and follow-up characteristics (n = 5077).

	Baseline	Four Years After Baseline	Eight Years After Baseline
Maternal age, mean (SD), years	37.3 (5.0)	41.4 (4.8)	45.4 (4.9)
Non-European ethnicity, n (%)	1955 (38.5)		
Educational level, n (%)			
No education/primary school	484 (9.5)		
Secondary school	2048 (40.3)		
Higher education	2545 (50.1)		
More than once pregnant, n (%)	4589 (90.4)		
Gravidity, median (90% range), n	3.0 (1.0; 9.2)		
BMI, median (90% range), kg/m^2^	24.4 (19.7; 35.3)	24.8 (20.1; 35.9)	25.1 (20.1; 35.9)
Current smoker, n (%)	1067 (21.0)		
PlGF, median (90% range), pg/mL	10.0 (6.0; 15.0)		
Systolic blood pressure, mean (SD), mmHg	119.4 (13.0)	114.5 (12.6)	117.2 (14.4)
Diastolic blood pressure, mean (SD), mmHg	71.0 (10.1)	68.5 (8.2)	70.3 (8.7)
Sustained hypertension, n (%)			435 (8.6)

Abbreviations: BMI, Body Mass Index; PlGF, placental growth factor. Values are presented as valid percentages for categorical variables, as means (SD) for continuous variables with a normal distribution, or as medians (90% range) for continuous variables with a skewed distribution. Imputed values are shown for confounders.

**Table 2:** Association of placental growth factor with cardiovascular parameters (n = 5077).

Exposure	Outcomes	
	**Baseline**	
PlGF (SDS) Beta (95% CI)	AOD, mm	−0.07 (−0.17; 0.04)
	LAD, mm	−0.06 (−0.18; 0.07)
	LV mass, g	−0.20 (−1.24; 0.83)
	PWV, m/s	0.00 (−0.04; 0.04)
	FS	0.14 (−0.04; 0.32)
	Central retinal arteriolar calibre (SDS)	−0.33 (−0.88; 0.23)
	Central retinal venular calibre (SDS)	0.54 (−0.21; 1.29)
	**Four years after baseline**	
	IMT, mm	0.00 (−0.00; 0.01)
	**Eight years after baseline**	
	PORH index	−0.92 (−2.00; 0.17)
	Sustained hypertension, OR (95% CI)	1.16 (1.01; 1.33) *

Abbreviations: SDS, standard deviation score; PlGF, placental growth factor; AOD, aortic root diameter; LAD, left atrial diameter; LV, left ventricular; PWV, pulse wave velocity; FS, fractional shortening; IMT, intima media thickness; PORH, post-occlusive reactive hyperaemia; CI, confidence interval. Values are regression coefficients with β and a 95% confidence interval, based on linear regression models. Outcomes are adjusted for maternal age, gravidity, ethnicity, educational level, smoking, and BMI at baseline. PWV is additionally adjusted for pulse at the time of PWV assessment. Arteriolar retinal calibre is additionally adjusted for venular calibre and vice versa. * *p*-value < 0.05.

**Table 3:** Association of placental growth factor measured at baseline with cardiovascular parameters in women with and without a placental syndrome in their index pregnancy.

	Women Without Placental Syndromes (n = 3608)	Women With Placental Syndromes (n = 756)
	PlGF (SDS) Beta (95% CI)	PlGF (SDS) Beta (95% CI)
**Baseline**		
PlGF, median (90% range), pg/mL	10.0 (6.0; 14.6)	10.0 (6.0; 16.0)
SBP (mmHg)	0.27 (−0.18; 0.72)	0.68 (−0.53; 1.88)
DBP (mmHg)	0.21 (−0.14; 0.56)	0.22 (−0.75; 1.19)
AOD, mm	−0.11 (−0.22; 0.01)	0.05 (−0.20; 0.31)
LAD, mm	−0.03 (−0.17; 0.10)	−0.08 (−0.39; 0.23)
LV mass, g	−0.91 (−2.05; 0.24)	2.72 (−0.02; 5.46)
PWV, m/s	0.00 (−0.04; 0.04)	−0.06 (−0.15; 0.04)
FS	0.11 (−0.09; 0.31)	0.05 (−0.44; 0.54)
Central retinal arteriolar calibre (SDS)	−0.57 (−1.19; 0.06)	0.65 (−0.75; 2.05)
Central retinal venular calibre (SDS)	0.74 (−0.12; 1.59)	−0.20 (−1.95; 1.55)
**Four years after baseline**		
SBP (mmHg)	0.43 (−0.04; 0.90)	−0.03 (−1.48; 1.42)
DBP (mmHg)	0.39 (0.05; 0.73) *	0.06 (−0.89; 1.00)
IMT, mm	0.00 (−0.00; 0.00)	0.00 (−0.01; 0.01)
**Eight years after baseline**		
SBP (mmHg)	0.75 (0.12; 1.37) *	0.62 (−1.10; 2.34)
DBP (mmHg)	0.41 (0.00; 0.81) *	0.30 (−0.77; 1.37)
PORH index	−1.16 (−2.51; 0.20)	−0.02 (−0.19; 0.15)
Sustained hypertension, OR (95% CI)	1.10 (0.93; 1.29)	1.20 (0.87; 1.65)

Abbreviations: SDS, standard deviation score; SBP, systolic blood pressure; DBP, diastolic blood pressure; PlGF, placental growth factor; AOD, aortic root diameter; LAD, left atrial diameter; LV, left ventricular; PWV, pulse wave velocity; FS, fractional shortening; IMT, intima media thickness; PORH, post-occlusive reactive hyperaemia; CI, confidence interval. Values are regression coefficients with β and a 95% confidence interval, based on linear regression models. Outcomes are adjusted for maternal age, gravidity, ethnicity, educational level, smoking, and BMI at baseline. PWV is additionally adjusted for pulse at the time of PWV assessment. Arteriolar retinal calibre is additionally adjusted for venular calibre and vice versa. * *p*-value < 0.05.

## Data Availability

Data requests can be made to the secretary of Generation R.

## References

[1] Maglione D., Guerriero V., Viglietto G., Delli-Bovi P., Persico M.G. (1991). Isolation of a human placenta cDNA coding for a protein related to the vascular permeability factor. Proc. Natl. Acad. Sci. USA.

[2] Redman C.W., Staff A.C. (2015). Preeclampsia, biomarkers, syncytiotrophoblast stress, and placental capacity. Am. J. Obstet. Gynecol..

[3] Coolman M., Timmermans S., de Groot C.J.M., Russcher H., Lindemans J., Hofman A., Geurts-Moespot A.J., Sweep F.C.G.J., Jaddoe V.V.W., Steegers E.A.P. (2012). Angiogenic and Fibrinolytic Factors in Blood During the First Half of Pregnancy and Adverse Pregnancy Outcomes. Obstet. Gynecol..

[4] Bellamy L., Casas J.P., Hingorani A.D., Williams D.J. (2007). Pre-eclampsia and risk of cardiovascular disease and cancer in later life: Systematic review and meta-analysis. BMJ.

[5] Benschop L., Schalekamp-Timmermans S., Broere-Brown Z.A., Roeters van Lennep J.E., Jaddoe V.W.V., Roos-Hesselink J.W., Ikram M.K., Steegers E.A.P., Roberts J.M., Gandley R.E. (2019). Placental Growth Factor as an Indicator of Maternal Cardiovascular Risk After Pregnancy. Circulation.

[6] Luttun A., Tjwa M., Moons L., Wu Y., Angelillo-Scherrer A., Liao F., Nagy J.A., Hooper A., Priller J., De Klerck B. (2002). Revascularization of ischemic tissues by PlGF treatment, and inhibition of tumor angiogenesis, arthritis and atherosclerosis by anti-Flt1. Nat. Med..

[7] Hoffmann D.C., Willenborg S., Koch M., Zwolanek D., Muller S., Becker A.K., Metzger S., Ehrbar M., Kurschat P., Hellmich M. (2013). Proteolytic processing regulates placental growth factor activities. J. Biol. Chem..

[8] Heeschen C., Dimmeler S., Fichtlscherer S., Hamm C.W., Berger J., Simoons M.L., Zeiher A.M., Investigators C. (2004). Prognostic value of placental growth factor in patients with acute chest pain. JAMA.

[9] Cianfarani F., Zambruno G., Brogelli L., Sera F., Lacal P.M., Pesce M., Capogrossi M.C., Failla C.M., Napolitano M., Odorisio T. (2006). Placenta growth factor in diabetic wound healing: Altered expression and therapeutic potential. Am. J. Pathol..

[10] Maes C., Coenegrachts L., Stockmans I., Daci E., Luttun A., Petryk A., Gopalakrishnan R., Moermans K., Smets N., Verfaillie C.M. (2006). Placental growth factor mediates mesenchymal cell development, cartilage turnover, and bone remodeling during fracture repair. J. Clin. Investig..

[11] Oura H., Bertoncini J., Velasco P., Brown L.F., Carmeliet P., Detmar M. (2003). A critical role of placental growth factor in the induction of inflammation and edema formation. Blood.

[12] Van de Veire S., Stalmans I., Heindryckx F., Oura H., Tijeras-Raballand A., Schmidt T., Loges S., Albrecht I., Jonckx B., Vinckier S. (2010). Further pharmacological and genetic evidence for the efficacy of PlGF inhibition in cancer and eye disease. Cell.

[13] Kim K.J., Cho C.S., Kim W.U. (2012). Role of placenta growth factor in cancer and inflammation. Exp. Mol. Med..

[14] Dewerchin M., Carmeliet P. (2012). PlGF: A multitasking cytokine with disease-restricted activity. Cold Spring Harb. Perspect. Med..

[15] Carmeliet P., Moons L., Luttun A., Vincenti V., Compernolle V., De Mol M., Wu Y., Bono F., Devy L., Beck H. (2001). Synergism between vascular endothelial growth factor and placental growth factor contributes to angiogenesis and plasma extravasation in pathological conditions. Nat. Med..

[16] Gigante B., Tarsitano M., Cimini V., De Falco S., Persico M.G. (2004). Placenta growth factor is not required for exercise-induced angiogenesis. Angiogenesis.

[17] Marrony S., Bassilana F., Seuwen K., Keller H. (2003). Bone morphogenetic protein 2 induces placental growth factor in mesenchymal stem cells. Bone.

[18] Fischer C., Mazzone M., Jonckx B., Carmeliet P. (2008). FLT1 and its ligands VEGFB and PlGF: Drug targets for anti-angiogenic therapy?. Nat. Rev. Cancer.

[19] Lenderink T., Heeschen C., Fichtlscherer S., Dimmeler S., Hamm C.W., Zeiher A.M., Simoons M.L., Boersma E., Investigators C. (2006). Elevated placental growth factor levels are associated with adverse outcomes at four-year follow-up in patients with acute coronary syndromes. J. Am. Coll. Cardiol..

[20] Tarnow L., Astrup A.S., Parving H.H. (2005). Elevated placental growth factor (PlGF) predicts cardiovascular morbidity and mortality in type 1 diabetic patients with diabetic nephropathy. Scand. J. Clin. Lab. Investig. Suppl..

[21] Cassidy A., Chiuve S.E., Manson J.E., Rexrode K.M., Girman C.J., Rimm E.B. (2009). Potential role for plasma placental growth factor in predicting coronary heart disease risk in women. Arter. Thromb. Vasc. Biol..

[22] Khurana R., Moons L., Shafi S., Luttun A., Collen D., Martin J.F., Carmeliet P., Zachary I.C. (2005). Placental growth factor promotes atherosclerotic intimal thickening and macrophage accumulation. Circulation.

[23] Kooijman M.N., Kruithof C.J., van Duijn C.M., Duijts L., Franco O.H., van I.M.H., de Jongste J.C., Klaver C.C., van der Lugt A., Mackenbach J.P. (2016). The Generation R Study: Design and cohort update 2017. Eur. J. Epidemiol..

[24] Kruithof C.J., Kooijman M.N., van Duijn C.M., Franco O.H., de Jongste J.C., Klaver C.C.W., Mackenbach J.P., Moll H.A., Raat H., Rings E.H.H.M. (2014). The Generation R Study: Biobank update 2015. Eur. J. Epidemiol..

[25] Devereux R.B., Alonso D.R., Lutas E.M., Gottlieb G.J., Campo E., Sachs I., Reichek N. (1986). Echocardiographic assessment of left ventricular hypertrophy: Comparison to necropsy findings. Am. J. Cardiol..

[26] Knudtson M.D., Lee K.E., Hubbard L.D., Wong T.Y., Klein R., Klein B.E. (2003). Revised formulas for summarizing retinal vessel diameters. Curr. Eye Res..

[27] Coolman M., de Groot C.J.M., Jaddoe V.W., Hofman A., Raat H., Steegers E.A.P. (2010). Medical record validation of maternally reported history of preeclampsia. J. Clin. Epidemiol..

[28] Brown M.A., Lindheimer M.D., de Swiet M., Van Assche A., Moutquin J.M. (2001). The classification and diagnosis of the hypertensive disorders of pregnancy: Statement from the International Society for the Study of Hypertension in Pregnancy (ISSHP). Hypertens. Pregnancy.

[29] Graham J.W., Olchowski A.E., Gilreath T.D. (2007). How many imputations are really needed? Some practical clarifications of multiple imputation theory. Prev. Sci..

[30] Zera C.A., Seely E.W., Wilkins-Haug L.E., Lim K.H., Parry S.I., McElrath T.F. (2014). The association of body mass index with serum angiogenic markers in normal and abnormal pregnancies. Am. J. Obstet. Gynecol..

[31] Pervanidou P., Chouliaras G., Akalestos A., Bastaki D., Apostolakou F., Papassotiriou I., Chrousos G.P. (2014). Increased placental growth factor (PlGF) concentrations in children and adolescents with obesity and the metabolic syndrome. Hormones.

[32] Krauss T., Pauer H.U., Augustin H.G. (2004). Prospective analysis of placenta growth factor (PlGF) concentrations in the plasma of women with normal pregnancy and pregnancies complicated by preeclampsia. Hypertens. Pregnancy.

[33] Chau K., Hennessy A., Makris A. (2017). Placental growth factor and pre-eclampsia. J. Hum. Hypertens..

[34] Levine R.J., Maynard S.E., Qian C., Lim K.H., England L.J., Yu K.F., Schisterman E.F., Thadhani R., Sachs B.P., Epstein F.H. (2004). Circulating angiogenic factors and the risk of preeclampsia. N. Engl. J. Med..

[35] Powers R.W., Roberts J.M., Plymire D.A., Pucci D., Datwyler S.A., Laird D.M., Sogin D.C., Jeyabalan A., Hubel C.A., Gandley R.E. (2012). Low placental growth factor across pregnancy identifies a subset of women with preterm preeclampsia: Type 1 versus type 2 preeclampsia?. Hypertension.

[36] Higashi Y., Kihara Y., Noma K. (2012). Endothelial dysfunction and hypertension in aging. Hypertens. Res..

[37] Pilarczyk K., Sattler K.J.E., Galili O., Versari D., Olson M.L., Meyer F.B., Zhu X.Y., Lerman L.O., Lerman A. (2008). Placenta growth factor expression in human atherosclerotic carotid plaques is related to plaque destabilization. Atherosclerosis.

[38] Shamim-Uzzaman Q.A., Pfenninger D., Kehrer C., Chakrabarti A., Kacirotti N., Rubenfire M., Brook R., Rajagopalan S. (2002). Altered cutaneous microvascular responses to reactive hyperaemia in coronary artery disease: A comparative study with conduit vessel responses. Clin. Sci..

[39] Lanting S.M., Barwick A.L., Twigg S.M., Johnson N.A., Baker M.K., Chiu S.K., Caterson I.D., Chuter V.H. (2017). Post-occlusive reactive hyperaemia of skin microvasculature and foot complications in type 2 diabetes. J. Diabetes Complicat..

[40] Huri M., Abati I., Bartolini C., Piacenza A., Tofani L., Vallario A., Di Tommaso M., Seravalli V. (2025). Correlation between first trimester placental growth factor levels and skin microvascular reactivity assessed by laser speckle contrast imaging—A cross-sectional study. Placenta.

[41] Sanip Z., Pahimi N., Bokti N.A., Yusof Z., Mohamed M.S., Isa W.Y.H.W., Rasool A.H. (2023). Impaired peripheral microvascular reactivity in patients with nonobstructive coronary artery disease. Microcirculation.

[42] Horton W.B., Barrett E.J. (2021). Microvascular Dysfunction in Diabetes Mellitus and Cardiometabolic Disease. Endocr. Rev..

[43] Xu C., Sellke F.W., Abid M.R. (2022). Assessments of microvascular function in organ systems. Am. J. Physiol. Heart Circ. Physiol..

[44] Elagizi A., Kachur S., Lavie C.J., Carbone S., Pandey A., Ortega F.B., Milani R.V. (2018). An Overview and Update on Obesity and the Obesity Paradox in Cardiovascular Diseases. Prog. Cardiovasc. Dis..

[45] Lavie C.J., Sharma A., Alpert M.A., De Schutter A., Lopez-Jimenez F., Milani R.V., Ventura H.O. (2016). Update on Obesity and Obesity Paradox in Heart Failure. Prog. Cardiovasc. Dis..

[46] Pascual M., Pascual D.A., Soria F., Vicente T., Hernandez A.M., Tebar F.J., Valdes M. (2003). Effects of isolated obesity on systolic and diastolic left ventricular function. Heart.

[47] Ikejder Y., Sebbani M., Hendy I., Khramz M., Khatouri A., Bendriss L. (2020). Impact of Arterial Hypertension on Left Atrial Size and Function. Biomed Res. Int..

[48] Landi F., Calvani R., Picca A., Tosato M., Martone A.M., Ortolani E., Sisto A., D’Angelo E., Serafini E., Desideri G. (2018). Body Mass Index is Strongly Associated with Hypertension: Results from the Longevity Check-up 7+ Study. Nutrients.

[49] Skoda M., Stangret A., Szukiewicz D. (2018). Fractalkine and placental growth factor: A duet of inflammation and angiogenesis in cardiovascular disorders. Cytokine Growth Factor Rev..

[50] Matsui M., Samejima K., Takeda Y., Tanabe K., Morimoto K., Okamoto K., Tagawa M., Onoue K., Okayama S., Kawata H. (2015). Prognostic Impact of Placental Growth Factor on Mortality and Cardiovascular Events in Dialysis Patients. Am. J. Nephrol..

[51] Bui A.H., Bonaca M.P., Sabatine M.S., Ray K.K., Rifai N., Cannon C.P., Morrow D.A. (2012). Elevated concentration of placental growth factor (PlGF) and long term risk in patients with acute coronary syndrome in the PROVE IT-TIMI 22 trial. J. Thromb. Thrombolysis.

[52] Santalahti K., Havulinna A., Maksimow M., Zeller T., Blankenberg S., Vehtari A., Joensuu H., Jalkanen S., Salomaa V., Salmi M. (2017). Plasma levels of hepatocyte growth factor and placental growth factor predict mortality in a general population: A prospective cohort study. J. Intern. Med..

[53] Chen Y., Nilsson A.H., Goncalves I., Edsfeldt A., Engstrom G., Melander O., Orho-Melander M., Rauch U., Tengryd C., Venuraju S.M. (2020). Evidence for a protective role of placental growth factor in cardiovascular disease. Sci. Transl. Med..

[54] Folkersen L., Gustafsson S., Wang Q., Hansen D.H., Hedman Å.K., Schork A., Page K., Zhernakova D.V., Wu Y., Peters J. (2020). Genomic evaluation of circulating proteins for drug target characterisation and precision medicine. bioRxiv.

[55] Nucci M., Poon L.C., Demirdjian G., Darbouret B., Nicolaides K.H. (2014). Maternal serum placental growth factor (PlGF) isoforms 1 and 2 at 11–13 weeks’ gestation in normal and pathological pregnancies. Fetal Diagn. Ther..

[56] De Falco S. (2012). The discovery of placenta growth factor and its biological activity. Exp. Mol. Med..

[57] Yang W., Ahn H., Hinrichs M., Torry R.J., Torry D.S. (2003). Evidence of a novel isoform of placenta growth factor (PlGF-4) expressed in human trophoblast and endothelial cells. J. Reprod. Immunol..

[58] Platt J.L., Vercellotti G.M., Lindman B.J., Oegema T.R., Jr Bach F.H., Dalmasso A.P. (1990). Release of heparan sulfate from endothelial cells. Implications for pathogenesis of hyperacute rejection. J. Exp. Med..

[59] Poon L.C., Zaragoza E., Akolekar R., Anagnostopoulos E., Nicolaides K.H. (2008). Maternal serum placental growth factor (PlGF) in small for gestational age pregnancy at 11(+0) to 13(+6) weeks of gestation. Prenat. Diagn..

[60] Akolekar R., Zaragoza E., Poon L.C., Pepes S., Nicolaides K.H. (2008). Maternal serum placental growth factor at 11 + 0 to 13 + 6 weeks of gestation in the prediction of pre-eclampsia. Ultrasound Obstet. Gynecol..

[61] Lecarpentier É., Vieillefosse S., Haddad B., Fournier T., Leguy M.C., Guibourdenche J., Tsatsaris V. (2016). Placental growth factor (PlGF) and sFlt-1 during pregnancy: Physiology, assay and interest in preeclampsia Le facteur de croissance placentaire (PlGF) et son récepteur soluble (sFlt-1) au cours de la grossesse: Physiologie, dosage et intérêt dans la préeclampsie. Ann. Biol. Clin..

[62] Umapathy A., Chamley L.W., James J.L. (2020). Reconciling the distinct roles of angiogenic/anti-angiogenic factors in the placenta and maternal circulation of normal and pathological pregnancies. Angiogenesis.

[63] Netea R.T., Smits P., Lenders J.W.M., Thien T. (1998). Does it matter whether blood pressure measurements are taken with subjects sitting or supine?. J. Hypertens..

[64] Lu L.C., Wei T.M., Li S., Ye X.L., Zeng C.L., Wang L.X. (2008). Differences in blood pressure readings between supine and sitting positions in hypertensive patients. Acta Cardiol..

[65] Jacobsen T.N., Nohr E.A., Frydenberg M. (2010). Selection by socioeconomic factors into the Danish National Birth Cohort. Eur. J. Epidemiol..

